# Epidemic spreading in multiplex networks influenced by opinion exchanges on vaccination

**DOI:** 10.1371/journal.pone.0186492

**Published:** 2017-11-09

**Authors:** Lucila G. Alvarez-Zuzek, Cristian E. La Rocca, José R. Iglesias, Lidia A. Braunstein

**Affiliations:** 1 Instituto de Investigaciones Físicas de Mar del Plata (IFIMAR-CONICET), Facultad de Ciencias Exactas y Naturales, Universidad Nacional de Mar del Plata, Déan Funes 3350, Mar del Plata, Argentina; 2 Programa de Pós-Graduação em Economia, Escola de Gestão e Negócios, UNISINOS, 93022-000, São Leopoldo, RS, Brazil; 3 Instituto Nacional de Ciência e Tecnologia de Sistemas Complexos, CBPF, Rio de Janeiro, RJ, Brazil; Universidad de Zaragoza, SPAIN

## Abstract

Through years, the use of vaccines has always been a controversial issue. People in a society may have different opinions about how beneficial the vaccines are and as a consequence some of those individuals decide to vaccinate or not themselves and their relatives. This attitude in face of vaccines has clear consequences in the spread of diseases and their transformation in epidemics. Motivated by this scenario, we study, in a simultaneous way, the changes of opinions about vaccination together with the evolution of a disease. In our model we consider a multiplex network consisting of two layers. One of the layers corresponds to a social network where people share their opinions and influence others opinions. The social model that rules the dynamic is the M-model, which takes into account two different processes that occurs in a society: persuasion and compromise. This two processes are related through a parameter *r*, *r* < 1 describes a moderate and committed society, for *r* > 1 the society tends to have extremist opinions, while *r* = 1 represents a neutral society. This social network may be of real or virtual contacts. On the other hand, the second layer corresponds to a network of physical contacts where the disease spreading is described by the SIR-Model. In this model the individuals may be in one of the following four states: Susceptible (*S*), Infected(*I*), Recovered (*R*) or Vaccinated (*V*). A Susceptible individual can: i) get vaccinated, if his opinion in the other layer is totally in favor of the vaccine, ii) get infected, with probability *β* if he is in contact with an infected neighbor. Those *I* individuals recover after a certain period *t*_*r*_ = 6. Vaccinated individuals have an extremist positive opinion that does not change. We consider that the vaccine has a certain effectiveness *ω* and as a consequence vaccinated nodes can be infected with probability *β*(1 − *ω*) if they are in contact with an infected neighbor. In this case, if the infection process is successful, the new infected individual changes his opinion from extremist positive to totally against the vaccine. We find that depending on the trend in the opinion of the society, which depends on *r*, different behaviors in the spread of the epidemic occurs. An epidemic threshold was found, in which below β* and above ω* the diseases never becomes an epidemic, and it varies with the opinion parameter *r*.

## Introduction

In 1796 Edward Jenner invented and tested a vaccine against the smallpox, an illness that had a very high index of mortality in the 18TH century [1]. The idea of Jenner was so successful that nowadays smallpox is practically eradicated and after this pioneering essay different vaccines were elaborated to prevent a long list of infectious diseases, from poliomyelitis to influenza. However, vaccines may present some lacks of efficiency and also some collateral effects. For example, in recent years some publications wrongly associated vaccination with autism [2, 3]. In spite of the overwhelming scientific evidence that such correlation is not actual, the belief that the results of vaccination could be worse than the illness itself spread through social networks and generated groups and movements against vaccination. Sometimes these groups are also related to some religion beliefs and/or rightists political tendencies or candidates. The debate about the efficiency of vaccination and its possible risks is then a very actual debate and a typical example of propagation of opinions, for and against vaccination. Thus, considering that opinions and contagion spreads in different ways, we will perform this study on a Network on Network. In recent years the study of complex Network of Networks (NoN) has been a subject of great interest for the scientific community, due to the large number of real word systems that can be mimic and study using these kind of topological structures [4–7]. A NoN is a system formed by single networks interacting through external connections between them. Many researches on NoN were focused in the study of cascade of failures [8–10], propagation of epidemics [11–14], and opinion dynamics [15–19] due to the ubiquitous of these processes that are present in the real scenarios. In particular, we are interested in processes that develop on NoN in which nodes belonging to different networks represents the same entities. This type of NoN are usually called *multiplex* networks. Epidemic spreading models have been particularly successful in understanding and predicting an epidemic outbreak and its period of extinction. Also, some models have incorporated a factor of human behavior, by considering the information and sources of information that individuals must handle, rational decisions and behavioral changes, in order to reach a more comprehensive understanding about the epidemic spreading [20]. A commonly-used model for reproducing spreading diseases dynamics in networks is the susceptible-infected-recovered (SIR) model [21–25]. This model has been successfully used to reproduce non recurrent diseases such as the H5N5 flu or the Severe Acute Respiratory Syndrome (SARS) [26]. Besides, it has been extensively studied under the topology of multiplex networks [27–30]. The model groups the population of individuals to be studied into three compartments according to their state: the susceptible (*S*), the infected (*I*), and the recovered (*R*). When a susceptible node is in contact with an infected node it becomes infected with an intrinsic probability *β*, which we called the virulence of the disease, and after a period of time *t*_*r*_ it recovers and becomes immune. Usually, the type of disease that this model describes has a period of infection that lasts for six or seven days on average, flu, for example.

The study of these models in real and synthetic networks [31, 32] have allowed researchers to develop different mitigation strategies for decreasing the impact of diseases on healthy populations [33–36]. These studies have been used in government policies to design vaccination campaigns. For instance, for seasonal diseases, such as influenza, vaccination campaigns are scheduled to begin before the epidemic spreads and in general this strategy is very effective [36]. Another strategy of prevention is the isolation for a certain period of time of individuals with infectious symptoms to prevent the spreading [34, 35]. Note that these scenarios are particularly interesting for epidemics spreading and the question that motivates this work is how the spread of the disease is influenced and co-evolves with the social context. Within the context of social phenomena, many empirical investigations show the importance of social influence in the formation of people’s opinions. It is argued that two interacting partners may exert social pressure to change their attitudes approaching their opinions [37]. This particular social mechanism is named *compromise* [15, 38–40]. A less explored mechanism of social interactions is the *persuasion* [41–44]. Myers [41] observed in group discussion experiments that when two individuals talk, they do not only state their opinions, but they also discuss about the arguments that support their opinions. If they hold the same opinion, they could strength it by persuading each other with new arguments or reasons, becoming more extreme in their believes. In this context, La Rocca *et. al* [45] proposed and studied a model that explains the phenomena of polarization in a population of individuals that evolve under pairwise interactions, by implementing those two main social mechanisms of opinion’s formation, i.e., compromise and persuasion [44, 46, 47]. This model, denoted as the M-model, has 2*M* different states describing the spectrum of possible opinion orientations on a given issue, from totally against (state *x* = −*M*) to totally in favor (*x* = *M*), with some moderate opinions between these extreme values.

The study of opinion dynamics on NoN is relatively new [6]. Alvarez-Zuzek *et al*. [48] investigated the interaction between two social dynamics, one for opinion formation and the other for decision making, on two interconnected networks. The dynamics for opinion formation corresponds to the M-model proposed in [45], and the decision making dynamics is akin to the Abrams-Strogatz (AS) model [49, 50] originally introduced to study language competition, where agents can choose between only two possible options (*x* = ±1). In this model each agent may change its decision by a mechanism of social pressure, in which the probability of switching his present choice increases non-linearly with the number of neighbors that have the opposite opinion. They concluded that under certain parameters of the system, one model prevails over the other and dominates the behavior of the system.

The goal of the present contribution is to investigate the effect of the dynamic of opinion formation on vaccination on the evolution of a given disease, for instance the flu. Thus, we will study the propagation of a disease in a population where all the individuals are continuously debating about getting vaccinated, considering that a susceptible individual is vaccinated if he is completely convinced about the benefits of the vaccine. However if after being vaccinated he catch the disease he becomes completely against the vaccination. For this purpose, and because the two processes occur on the same group of individuals, we studied the SIR model with vaccination and the M-model in a multiplex system composed by two networks. Both dynamics take place in different layers and co-evolve. Susceptible individuals become vaccinated if they acquire the state *M* in the other network, while the vaccinated individuals acquire the state −*M* if they get infected.

The paper is organized as follows: in the next section we expose the model presented in its extended form. In Section 3, we present the simulation results and Section 4 is devoted to discussion and conclusions.

## The model

We are interested in studying how the propagation of diseases is influenced by the opinion formation of individuals in favor or against of getting vaccinated. The opinions will be formed and/or modified through the interaction and exchange of ideas with other individuals, which have their own opinion and co-evolves with the health condition of those individuals. In this way, the group of individuals develop a dynamic of formation of opinions in which individuals interact expressing opinions about the importance or not of being vaccinated. When an individual has a fully positive opinion about the vaccine, he acts accordingly and gets vaccinated. In our model we do not consider parental decisions on children, so the opinion on vaccination motivates just individuals, not family groups. While the process of spreading a disease generally requires face-to-face physical contact, the process of formation of opinions is more flexible because opinions can be transmitted via other media: phone calls, online social networks, video conference and instant messaging services, etc.

To model the spread of the disease in layer *A* we use a variation of the SIR model in which a new stage of healthy vaccinated individual (*V*) is incorporated. Let’s recall that vaccinated individuals share opinion *M* = +2. i) An individual *S* in contact with an infected individual *I* becomes infected with a probability *β* (the infectivity of the disease). ii) However, as the vaccine does not guarantee 100% protection a vaccinated individual (*V*) can become infected with a probability (1 − *ω*)*β*, where *ω* is the efficiency of the vaccine. iii) An infected individual recovers after a period of time *t*_*r*_, and we assume he acquires immunity. If the vaccinated agent gets infected, he changes to the opposite opinion becoming an extremist against the vaccine (*M* = −2). This may be an extreme behavior and probably people are not so extremists, but our objective here is to describe the frustration of a vaccinated agent after acquiring the infection. Notice that, if we let this frustrated agent go to an intermediate opinion, the influence on the epidemics evolution is almost irrelevant.

For the process of opinion formation in network *B* we use the *M* model [45]. This model explains the phenomena of polarization in a population of interacting individuals and two main processes are involved: compromise and persuasion. We consider *M* = 2, being *M* (totally in favor) and −*M* (totally against) the extremist cases and the intermediate cases correspond to the states of moderate opinion. In our model we considered that only one individual, the *i*–agent, can change his opinion, assuming that the other one, the *j*–agent has enough arguments to convince or change the opinion state of the first individual. Then, the rules of the opinion model are:

A node *i* is chosen and it can change its opinion state after interaction with a neighbor *j*. If their respectively opinion states are *x*_*i*_ and *x*_*j*_, we proceed as follows:

- If both individuals have the same opinion orientation (i.e. *x*_*i*_*x*_*j*_ > 0), then with probability **p**: *x*_*i*_ = ±1 → *x*_*i*_ = ±2.
  In case that *i* is already in an extremist state (*x*_*i*_ ± 2) it remains extremist.
- If both individuals have different opinion orientations (i.e. *x*_*i*_ *x*_*j*_ < 0), then with probability **q**
  *x*_*i*_ = ±1 → *x*_*i*_ = ∓1,
  *x*_*i*_ = ±2 → *x*_*i*_ = ±1.
  However, one assumes that if node *i* is a vaccinated agent, he keeps his opinion (and his vaccinated state) even when interacting with neighbor *j* having an opposite sign opinion.

If two nodes have the same opinion orientation, one of them becomes more extremist with probability *p*, but if they have different opinion orientations one of the individuals becomes more moderate with probability *q*. For simplicity, we consider *p* + *q* = 1 and define the ratio *r* = *p*/*q*. In our model an individual *S* becomes *V* if in layer *B* he reaches opinion 2. On the other hand, if an individual *V* becomes *I* with probability *β*(1 − *ω*), then in layer *B* he changes his opinion to −2. Notice that even when the recovered individuals becomes inactive in the layer *A*, they are still active in the layer *B*.

**Fig 1:**
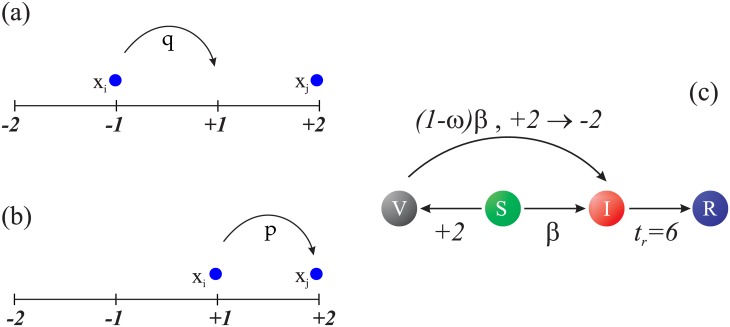
Simplified scheme of opinions and epidemics dynamics. Left figures illustrate the opinion dynamics, when two nodes have opinion states with different sign, one of them approaches its state to the opinion of the other with a probability *q* (a), whereas if the sign is the same the node reinforces its opinion with probability *p* (b). Right figure illustrate the contagion dynamics, a susceptible individual *S* (green) is infected (red) with probability *β* and after a time *t*_*r*_ he recovers (blue). A *S* becomes vaccinated *V* (gray) when he acquires a state of opinion 2, but then he can become *I* with a probability (1 − *ω*)*β*, changing his opinion to −2.

In Fig 1 we show a scheme of the rules of the dynamics of the whole system. An individual with opinion state *x*_*i*_ whose neighbor with state *x*_*j*_ has an opinion with different orientation approaches to the opinion of the neighbor with probability *q* (Fig 1a)), whereas if both individuals have the same orientation of opinion i-agent reinforces his opinion with probability *p* (Fig 1b)). Concerning the contagion (Fig 1c)), an individual *S* (green) becomes *I* (red) with a probability *β* and after a time *t*_*r*_ goes to the recovered state *R* (blue). An agent *S* is vaccinated *V* (gray) when acquiring the opinion state 2, and in contact with an infected individual can get infected with a probability (1 − *ω*)*β*, where *ω* (0 ≤ *ω* ≤ 1) is the efficiency of the vaccine. If the *V* agent is infected he loses its confidence in the vaccine and thereby changes his state of opinion from 2 to −2.

## Simulation results

We study the model described in the previous section by means of extensive Monte Carlo simulations with synchronous update using a two-layer network of the same size *N* = 10^5^. Nodes in each layer represent the same agent, thus we connect through an external link a pair of nodes, each from different layer, allowing only one interlink by node. We construct each layer using the Molloy-Reed algorithm [51] considering the Erdős-Rényi (ER) [52] degree distribution with 〈*k*〉 = 4. The propagation of the disease takes place in layer *A* and we fix the recovery time in *t*_*r*_ = 6, which is in days the characteristic period of infection for a flu. Layer *B* is the social network, where the M-model rules the dynamic, with *M* = 2. As initial conditions we use for the layer *B* an uniform distribution for the densities of opinion, i.e., the same initial probability *P*_+2,+1,−1,−2_ = 1/4. In layer *A* we have initially only one agent infected, which is considered the patient zero and whose opinion is chosen at random between the four possible opinion states, a fraction 1/4 of the agents are vaccinated ones, as a consequence of their opinion state +2, and the rest are susceptible. We chose one source node of infection because this is the standard approach used by epidemiologists where most outbreaks starts with one person. At each time steps, we first let evolve the epidemic dynamic and then the opinion process. In layer A, we allow all the infected individuals to infect each one of their susceptible neighbors with probability *β* and the vaccinated neighbors with a probability (1 − *ω*)*β*. Then, in the opinion layer, we iterate over all the individuals and give each one of them the chance to interact with only one of its neighbors. This neighbor is chosen among those who can change the individual opinion. In case there is no neighbor that can change the opinion, nothing happen. Finally, we update all the opinions and epidemic states at the next time step. Notice that those infected individuals who had *t*_*r*_ time step to spread the disease recover and those susceptible individuals whose opinion change to +2 change into the vaccinated state. All numerical results correspond to an average over 10^5^ independent realizations.

We concentrate in the steady state of the system which is reached when the number of infected nodes becomes zero, regardless of whether consensus was reached in the opinion network. Then, the magnitudes to be studied are the fraction of recovery nodes (*R*), the fraction of vaccinated nodes (*V*), the duration time of the epidemic (*τ*) and the magnetization of the opinions (*Mag*). Notice that at any time *S* + *I* + *R* + *V* = 1 and *Mag* = *σ*_+_ − *σ*_−_, where *σ*_±_ is the fraction of nodes with positive (negative) opinion state. We only take into account those realizations in which an epidemic occurs, i.e., the total number of recovered individuals in the final state is greater than a cutoff 200 for a system size of *N* = 10^5^ [53].

**Fig 2:**
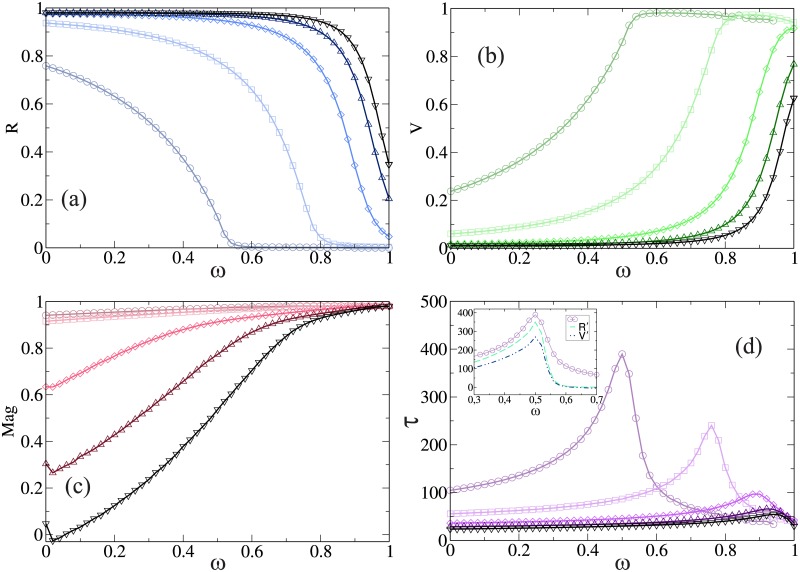
(a) Fraction of recovery individuals *R*, (b) Fraction of vaccinated individuals *V*, (c) Magnetization of the opinions *Mag* and (d) The duration time of the epidemic *τ*, as a function of the efficiency of the vaccine *ω*. Inset: *τ* (solid line), the derivative of *R* (dashed line) and the derivative of *V* (dot dashed line) as a function of the efficiency *ω* and *β* = 0.1. From the inset it is clear that the maximum duration of the epidemics corresponds to inflection points in the number of recovered and vaccinated agents. In all cases we set *t*_*r*_ = 6 and *r* = 0.1 for *β* = 0.1 (○), 0.2 (□), 0.4 (◇), 0.6 (△) and 0.8 (▽). All numerical results correspond to an average over 10^5^ independent realizations.

Now, we will present in further details *R*, *V*, *τ* and *Mag* as a function of the efficiency of the vaccine *ω*, for different values of the parameters *r* and *β*. In Fig 2 we present the case *r* = 0.1, that mimics a scenario in which the population’s opinion settles in a centralized state where the compromise process dominates.

In Fig 2(a) we show the total fraction of recovery nodes as a function of *ω* for different values of *β*. We can observe that for certain values of *β*, as *ω* increases the fraction *R* decreases and above a value ω*, which is a threshold for the efficiency of the vaccine, the system does not present an epidemic phase and corresponds to the inflection point of the curve. This is because as the vaccine becomes more effective, more people remain vaccinated and the propagation of the disease slows down. An efficiency above the threshold *ω* > ω* is enough to ensure that an epidemic will not develop, such as the case of low values of *β*. For example for *β* = 0.1 we need an efficiency of at least 55% in order to avoid the epidemic. On the other hand, above a certain value of β* the propagation of the disease is enhanced and it is impossible to prevent an epidemic. Even for *ω* = 1 there will be a macroscopic number of recovery individuals in the steady state. This is the case for the values *β* = 0.4, 0.6 and 0.8. Fig 2(b) and 2(c) shows the fraction of vaccinated nodes and the magnetization of the opinions as a function of *ω*. For all the values of *β* we can see that both magnitudes increases with *ω*. This is consistent with the fact that as the vaccine becomes more efficient, more people will agree to get vaccinated.

For *r* = 0.1 the compromise is higher than the persuasion and as a consequence agents tend to have moderate opinions (in favor or against). However, when an agent is vaccinated, his opinion (2) remains fixed producing an attractive effect towards positive opinion and he will only change his opinion if he gets infected. As can see from Fig 2(c), for low values of efficiency *Mag* decreases as *β* increases, for example, for *β* = 0.8 agents opinion are in a polarized state (*Mag* = 0). This behavior is due to the fact that as *β* increases more vaccinated agents gets infected, so their opinion change from 2 to −2, which means that more people become extremist against the vaccine. On the other hand, for an efficiency close to one, the opinion of the system is in average almost completely in favor of the vaccine, reaching a consensus where all the agents have the same opinion. Because the efficiency of the vaccine is high the vaccinated agents stay pinned in the opinion +2, pushing all the agents to adopt their opinion. Notice that in this scenario the convergence time of both dynamics are similar, *i*.*e*, the time that it takes to the disease to propagates all over the population allows the people to reach consensus in favor of the vaccine.

In Fig 2(d) we can see the duration time of the epidemic as a function of *ω*. As we can observe as *ω* increases more nodes are vaccinated and as a consequence the duration of the disease increases. Around the threshold ω* the time of the epidemic exhibits a peak and then decreases rapidly. This is consistent with the fact that as *ω* increases the number of *R* decreases, which means that it is hard to spread the disease and therefore *τ* increases. The time of duration of the epidemic reaches a maximum at ω* and then decreases because the spreading of the disease is diminish (there is no epidemic). Note that for *ω* > ω* the majority of the agents in the system are vaccinated. We added an inset in Fig 2, (as well as in the following ones), comparing the duration of the epidemics with the derivatives of the number of recovered (*R*) and vaccinated (*V*) agents as a function of the efficiency. It is possible to see that there is an inflection point (a maximum in the derivatives) when the duration of the epidemics is maximum, meaning that the number of recovered and vaccinated agents increase at a lower rate when the efficacy of the vaccine is high than when the efficacy is low, going trough a maximum rate when the duration of the epidemics is maximum.

**Fig 3:**
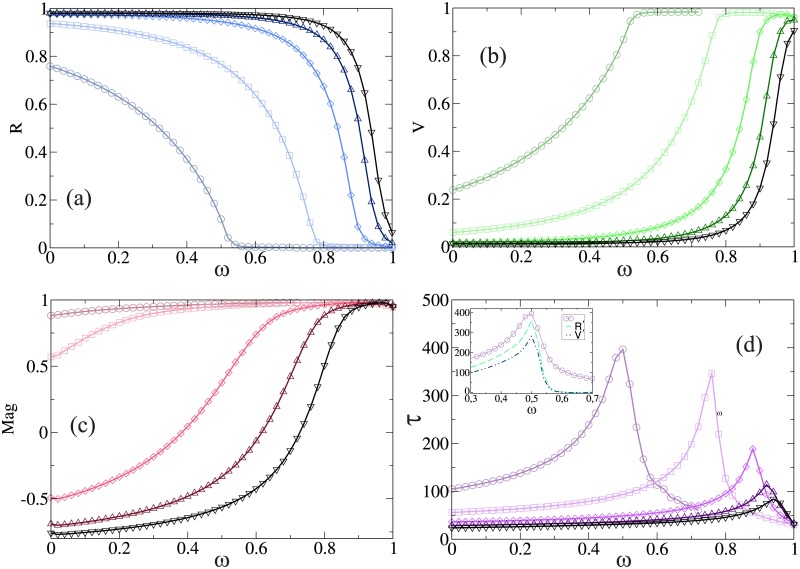
(a) Fraction of recovery individuals *R*, (b) Fraction of vaccinated individuals *V*, (c) Magnetization *Mag* and (d) The duration time of the epidemic *τ*, as a function of the efficiency of the vaccine *ω*. Inset: *τ* (solid line), the derivative of *R* (dashed line) and the derivative of *V* (dot dashed line) as a function of the efficiency *ω* and *β* = 0.1. In all cases we set *t*_*r*_ = 6 and *r* = 1 for the same values of *β* and symbols used in Fig 2. All numerical results correspond to an average over 10^5^ independent realizations.

In Fig 3 we show the case of *r* = 1, which mimics a neutral society where the probability of compromise and persuasion are equal (*p* = *q* = 0.5). Fig 3(a) shows *R* as a function *ω*. We can observe that an efficiency threshold exists (for low values of *β*) below which the diseases never becomes an epidemic. While for higher values of *β* there is always an epidemic phase. In Fig 3(c) we show *Mag* as a function of *ω*. From the plot we can see that for low values of *ω* the magnetization decreases as *β* increases, while for an efficiency close to one the system reaches consensus in favor of the vaccine. Besides, for low values of *ω* and high values of *β*, *Mag* becomes negative because of the persuasive effect, which is not negligible. Agents with negative opinion are less likely to change their opinion. Also, since the vaccine is not much effective, vaccinated agents gets infected, thus their opinions change to negative and this contributes to a negative magnetization. On the other hand, we can see that for large values of *ω* the convergence time of both dynamics are similar, the *Mag* in the opinion dynamic is close to one, thus is close to the consensus state. The opposite occurs for the other values of *ω*, where the model of opinions is far from consensus.

**Fig 4:**
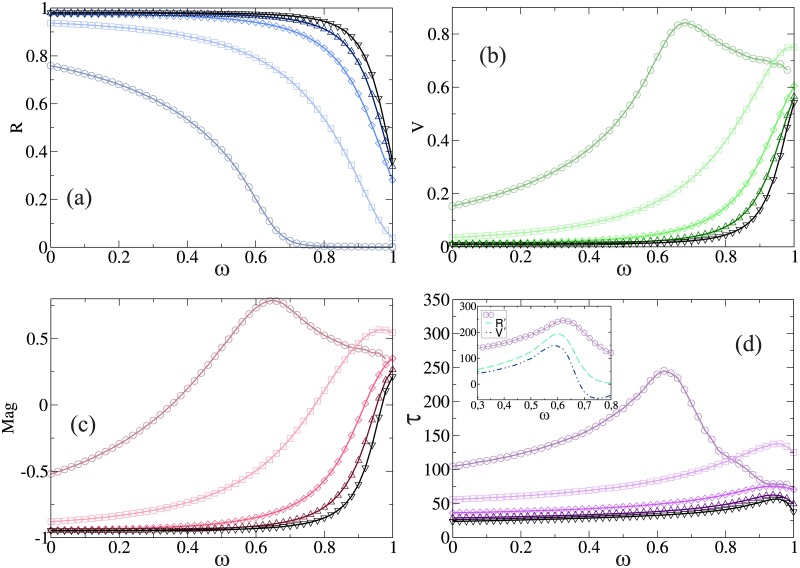
(a) Fraction of recovery individuals *R*, (b) Fraction of vaccinated individuals *V*, (c) Magnetization *Mag* and (d) The duration time of the epidemic *τ*, as a function of the efficiency of the vaccine *ω*. Inset: *τ* (solid line), the derivative of *R* (dashed line) and the derivative of *V* (dot dashed line) as a function of the efficiency *ω* and *β* = 0.1. In all cases we set *t*_*r*_ = 6 and *r* = 10 for the same values of *β* and symbols used in Fig 2. All numerical results correspond to an average over 10^5^ independent realizations.

Now we will show the case of *r* = 10, which represents an extremist society where persuasion dominates the process of opinion formation. In this case, agents with extremist opinion tend to convince agents with moderate opinion to become extremists. From Fig 4(a) and 4(d) we can see that the behavior of *R* and the time duration of the epidemic *τ* are qualitative the same that for the two previous cases studied of *r* with different β*. Agents become extremist in their opinions and those who are against the vaccine have a small probability to be vaccinated. Then the disease spreads is promoted among the non vaccinated agents, which are an important fraction of the population. In Fig 4(b) and 4(c) we can observe that *V* and *Mag* increase as *ω* increases, and for the cases below β* (as *β* = 0.1) both magnitudes increase with *ω* until reaching a peak around ω* after which these magnitudes decreases. This is due to the fact that above the point ω*, the time of the epidemic decreases as *ω* increases, and there is not enough time to convince the negative opinion agents to vaccinate.

As the persuasion is higher than compromise (*p* = 0.91 and *q* = 0.09), agents tend to remain with extremist opinions, against or in favor of the vaccine. The attractor effect that generate the vaccinated agents in the opinion state is now hidden by the persuasive effect. The persuasive effect moves agents to the extreme opinions. When a vaccinated agent is infected, its opinion becomes a negative extremist and in this extremist society he will rarely change his opinion. For this reason, for all values of *β* and low values of *ω* the *Mag* is always negative. On the other hand, for high values of the efficiency the average opinion of the system can be in favor or against depending of the virulence, but in general it is polarized and as a consequence, *Mag* closed to zero. Even for the case in which *ω* = 1 there still some agents that are against the vaccine. This is due to the fact that almost all agents that began with negative opinion remains in that state. Notice that the epidemic dynamics is faster than the dynamics of opinions -the convergence time is higher in layer *B*- making that the population never reach a consensus of opinions.

**Fig 5:**
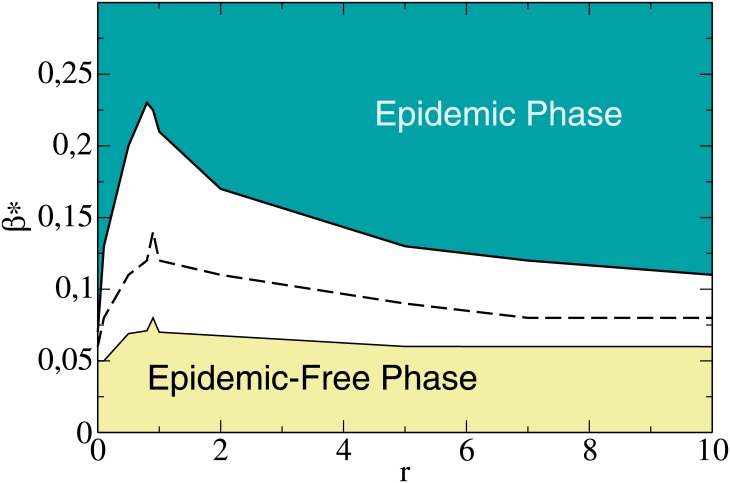
Threshold epidemic value β* as a function of *r*, with *t*_*r*_ = 6, *ω* = 1 and as initial conditions 1%, 10% and 25% of vaccinated nodes, from bottom to top. The maximum value is closed to *r* ≈ 1, which is a neutral society.

We mentioned before that there is a threshold β* above which the system always stay in an epidemic regime, independently of the efficiency of the vaccine, *ω*. In Fig 5 we show β* as a function of *r* for *t*_*r*_ = 6 and *ω* = 1. We set *ω* = 1, so that the vaccine is 100% effective, in order to know how strong the virulence of the disease has to be to win the best vaccination scenario. In addition, we also study this scenario for different values of initial vaccinated nodes -1%, 5% and 25%- to see how the initial conditions impacts on the evolution of both dynamics. As we can see from Fig 5, for a certain value of *r*, β* decreases as the initial vaccinated nodes decreases. This is consistent with the fact that having fewer initial vaccinated nodes causes the disease to spread more easily, so that less virulent diseases could become epidemic. As we can observe that the maximum values are around *r* ≈ 1, which means that a neutral society is optimal to prevent an epidemic. In a society with *r* << 1, compromise dominates the process of opinion formation and the agents tend to have a moderate opinion. This prevents those moderate agents from becoming extremists in favor of vaccination. The disease spreads through the non vaccinated agents very easily, even when the virulence is small. On the other hand, in a society with *r* >> 1, persuasion dominates the process of opinion formation and the agents tend to adopt extremists opinion. All extremists agents in favor of vaccination will be vaccinated, but those agents with a negative opinion (against the vaccine) have a small probability to be vaccinated because they will hardly change their opinion. In this case, the disease spreads very easily over these agents, which are an important fraction of the population. In a neutral society it is more likely for an agent with a positive moderate opinion to become an extremist in favor of the vaccine than in the case *r* << 1, and it is more likely for an agent against the vaccine to change his opinion in favor than in the case *r* >> 1. For this reason, β* is higher than in the other cases, because it is easier to convince people to get vaccinated, making more difficult for the disease to expand all over population.

## Discussion

In this paper, we studied the propagation of a disease in a population where all the individuals are continuously debating about getting vaccinated, considering that a susceptible individual is vaccinated if he is completely convinced about the benefits of the vaccine. For this purpose we used two-layer network where in one layer we use the SIR-model with vaccination for the propagation of a disease, and in the other layer we used the M-model (with *M* = 2), for the opinion formation process, where compromise and persuasion are the two processes involved and are controlled by the parameter *r*. We found that, in all the cases, the number of recovered agents decreases as *ω* increases, and this is due to the fact that as the vaccine becomes more effective, more people remain vaccinated and the propagation of the disease slows down. We found an epidemic threshold ω* above which we ensure that an epidemic will not develop. Furthermore, we found that above a certain value of β* the propagation of the disease is enhanced and it is impossible to prevent it from becoming an epidemic. Even for *ω* = 1 there will be a final macroscopic number of recovery individuals in the steady state. We computed this threshold as a function of *r*, and we found that a neutral society is the best scenario to prevent an epidemic (*r* ≈ 1). When compromise dominates the process of opinion formation (*r* << 1), the agents tend to have a moderate opinion, making difficult that they become extremist in favor of vaccination. The disease spreads through the non vaccinated agents very easily, even when the virulence is small. On the other hand, when persuasion dominates the process of opinion formation (*r* >> 1) the agents tend to have an extremist opinion. All the extremist agents in favor of the vaccine will be vaccinated, but those agents with a negative opinion, which are an important fraction of the population, will be easily infected. In a neutral society it is more likely to convince those agents with a negative opinion in favor on vaccination, to become extremist in favor. With compromise and persuasion in the same proportion it is easier to convince people to get vaccinated, blocking the propagation of the disease and preventing it to expand all over the population. We can conclude that the influence of the opinion on the vaccination determines, in certain cases, whether or not the disease becomes in an epidemic.
